# Intended or Unintended Doping? A Review of the Presence of Doping Substances in Dietary Supplements Used in Sports

**DOI:** 10.3390/nu9101093

**Published:** 2017-10-04

**Authors:** José Miguel Martínez-Sanz, Isabel Sospedra, Christian Mañas Ortiz, Eduard Baladía, Angel Gil-Izquierdo, Rocio Ortiz-Moncada

**Affiliations:** 1 Nursing Department, Faculty of Health Sciences, University of Alicante, 03690 Alicante, Spain; isospedra@ua.es; 2 Research Group on Food and Nutrition (ALINUT), University of Alicante, 03690 Alicante, Spain; e.baladia@academianutricion.org (E.B.); angelgil@cebas.csic.es (A.G.-I.); rocio.ortiz@ua.es (R.O.-M.); 3 Pharmacy faculty, University of Valencia, 46100 Valencia, Spain; christianmanas@hotmail.es; 4 Evidence-Based Nutrition Network (RED-NuBE), Spanish Academy of Nutrition and Dietetics (AEND), 31006 Navarra, Spain; 5 Quality, Safety, and Bioactivity of Plant Foods Group, Department of Food Science and Technology, CEBAS-CSIC, University of Murcia, 30100 Murcia, Spain; 6 Department of Community Nursing, Preventive Medicine and Public Health and History of Science Health, University of Alicante, 03690 Alicante, Spain

**Keywords:** dietary supplements, doping, ergonutritional aids, WADA

## Abstract

Introduction: The use of dietary supplements is increasing among athletes, year after year. Related to the high rates of use, unintentional doping occurs. Unintentional doping refers to positive anti-doping tests due to the use of any supplement containing unlisted substances banned by anti-doping regulations and organizations, such as the World Anti-Doping Agency (WADA). The objective of this review is to summarize the presence of unlabeled doping substances in dietary supplements that are used in sports. Methodology: A review of substances/metabolites/markers banned by WADA in ergonutritional supplements was completed using PubMed. The inclusion criteria were studies published up until September 2017, which analyzed the content of substances, metabolites and markers banned by WADA. Results: 446 studies were identified, 23 of which fulfilled all the inclusion criteria. In most of the studies, the purpose was to identify doping substances in dietary supplements. Discussion: Substances prohibited by WADA were found in most of the supplements analyzed in this review. Some of them were prohormones and/or stimulants. With rates of contamination between 12 and 58%, non-intentional doping is a point to take into account before establishing a supplementation program. Athletes and coaches must be aware of the problems related to the use of any contaminated supplement and should pay special attention before choosing a supplement, informing themselves fully and confirming the guarantees offered by the supplement.

## 1. Introduction

According to European Parliament Directive (2002/46/EC), a food supplement is defined as a product intended to supplement the normal diet, consisting of a concentrated source of a nutrient or of other substances that have a nutritional or physiological effect, in a simple or combined form, commercialized in dosed formulas, capsules, tablets, pills and other similar forms, bags of powder, vials of liquid, dropper bottles and other similar forms of liquids and powders, which is taken in small, quantified amounts [1]. In sport—understood as a set of motor situations codified in the form of competition, and institutionalized—athletes use ergogenic aids (any nutritional, physical, mechanical, psychological, or pharmacological maneuver or method) in order to increase their ability to perform physical work and improve performance [2]. In sport, dietary supplements (also known as ergonutritional aids) have been used since the first Olympic Games, although recently there has been a notable increase in their consumption by certain population groups [3,4,5]. Athletes consume a wide variety of dietary supplements and are the main target of the industry that produces them [6]. The “Sports Nutrition and Weight Loss Report”, published by the Nutrition Business Journal, showed that sales of sports nutrition and weight loss products have increased year-on-year in the North American market, with nutritional supplements being in second place in the sales ranking [7]. 

This indicates that the sale and consumption of supplements have increased both in the general population and in the sports population. In a study of 3168 British Royal Army soldiers, a rate of supplement use of 38% at the time of the study was reported, reaching 54% when the use of supplements referred to the 12 months prior to the study [8]. In order of prevalence, the most used supplements were: protein powders/bars (66%), isotonic sports drinks (49%), creatine (38%), recovery drinks (35%), multivitamins (31%), and Vitamin C (25%). The work of Tscholl et al. was performed on 3887 athletes, and found a total consumption of 6523 supplements (1.7 per athlete) [9].

Some athletes have been reported to have tested positive for doping due to the intake of dietary supplements, which had either poor labeling or product contamination [5,10]. This poses a threat to the athlete′s career or also to his or her health depending on the dose, as the World Anti-Doping Agency (WADA) states that it is the athlete′s responsibility to ensure no prohibited substance or its metabolite or marker are in the samples [11]. An example of the presence of doping substances in supplements can be seen in the study published in 2003 by Geyer et al., where 94 of the 634 supplements analyzed (14.8%) had prohormones that were not mentioned on the label [12]. More current is the study by Judkins et al. in which, of the 58 supplements analyzed, 25% contained low levels of contaminating steroids and 11% were contaminated with stimulants [13]. These data have led to the investigation of contamination in different food supplements; in most of them, small quantities of banned substances have been found, due to cross-contamination during manufacturing, processing, or packaging [14,15,16,17]. In some cases, this contamination was not intentional and was due to poor quality control, but in others the adulteration of the substance was intentional [10]. In the United States (US), the Food and Drug Administration (FDA), broadly speaking, regulates quality, and the Federal Trade Commission supervises the marketing and advertising of dietary supplements [18]. However, according to the Dietary Supplement Health and Education Act (DSHEA), dietary supplements, including nutritional ergogenic aids, that are not intended to diagnose, treat, cure, or prevent any disease, currently do not need to be evaluated by the FDA prior to their commercialization [19].

Despite the proposed legislation and the pressure exerted by governments [20,21] and various organizations, such as the WADA, through the list of prohibited substances and methods, or the International Olympic Committee (IOC), with the acceptance of the World Anti-Doping Code [22,23], positive tests continue to occur in anti-doping checks due to products containing prohibited substances that are not listed in their labeling. One example is 19-norandrosterone, a substance found alongside stimulants, such as caffeine and epinephrine, in certain dietary supplements [24]. In 2003, after observing a series of repeated cases, a study was performed to determine the extent of the problem of unidentified prohormones in dietary supplements, giving positive results for 19-norandrosterone with the intake of only one capsule of product, while the proposed dose is four capsules, three times a day [25]. More recently, in a review of 24 different types of protein supplement, carried out in 2010 by ConsumerLab, 31% of products did not pass the proposed safety test, leaving in question the supposed safety that these products offer the consumer [26]. 

Therefore, the objective of the present work is to describe the presence of doping substances prohibited by the WADA in dietary supplements, used in the context of sport and published in research articles, thereby highlighting the problem of plausible positive tests in anti-doping checks and the health problems that could be generated by their unintended consumption.

## 2. Materials and Methods

This is a descriptive study, consisting of a bibliographic review of the presence of substances/metabolites/markers prohibited by the WADA in dietary supplements used in the sporting context. Contamination is understood as introduction into a medium of substances that cause it to be unsafe or unfit for use; in our case, the incorporation and non-declaration in the labeling of substances/metabolites/markers prohibited by WADA into ergonutritional supplements used by athletes. A data collection protocol was established for the research that met the inclusion criteria. The screening of the articles was performed by two researchers, independently. 

A structured restrictive search was performed in the PubMed, Tripdatabase, and Epistemonikos databases using controlled and natural vocabulary descriptors related to “doping agents” and “Dietary supplements” concepts. The full electronic search strategy for PubMed was: (“prohibited substance” [tiab (title/abstract)] or “banned substance” [tiab] or “banned substances” [tiab] or “Doping in Sports” [Mesh] or “Doping in Sports” [tiab] or Doping [tiab] or “doping agent” [tiab] or “doping agents” [tiab]) AND (“Dietary Supplements” [Mesh] or “Dietary Supplements” [tiab] or “Dietary Supplement” [tiab] or Nutraceuticals [tiab] or Nutraceutical [tiab] or Nutriceutical [tiab] or Nutriceuticals [tiab] or Neutraceutical [tiab] or Neutraceuticals [tiab] or “Food Supplementations” [tiab] or “Food Supplementation” [tiab] or “Ergogenic aids” [tiab] or “Ergogenic aid” [tiab] or “dietary supplement, SPORT” [Supplementary Concept] or “nutritional supplement” [tiab] or “nutritional supplements” [tiab]). Also, relevant references related to the topic of the selected articles were searched for manually, using a snow-ball method. No additional filters were applied, and the last search was performed on 17 September 2017.

The eligibility criteria to select articles was:

- Evaluation of marketed dietary supplements for intended use in sports
- Evaluation of any type of prohibited substance, as defined by WADA (World Anti-Doping Agency. 2017 List of prohibited substances and methods. 2017 [22].)
- Only primary research was allowed, but secondary research was screened (by bibliography)
- No limits were set according language, years considered, or publication status or availability.

Two independent researchers screened titles and abstracts to pre-select studies from the list of articles retrieved by the search strategy. One researcher screened the pre-selected articles, by full-text reading, to apply eligibility criteria and a second researcher reviewed the selections, to ensure that all studies should be included. One researcher performed the data extraction without piloted forms, but a second researcher reviewed the extracted data to avoid extraction mistakes or missing information. 

From selected studies, the extracted data set was composed of the following variables:

- Author/year: authors and year of publication.
- Country: geographical area from which the results obtained in the study come.
- Aim of the study: results that were intended to be achieved with the study.
- Sample: number and type of supplements analyzed.
- Methodology for the analysis of banned substances/metabolites/markers.
- Selected markers: tested substances/metabolites/markers that give positive results in anti-doping controls.
- Main results: final outcomes of the study, in which it is shown whether the proposed objectives have been achieved, and the main results obtained are listed.
- Conclusions: arguments and statements concerning the data obtained in the studies.

No risk of bias analysis in the included studies was performed and data was summarized through table summaries. No additional analysis was performed.

## 3. Results

The search strategy retrieved 446 articles (PubMed *n* = 378; Tripdatabase *n* = 67; Epistemonikos *n* = 0; 1 added manually from a review screening), which resulted in 423 unique articles after duplicate removal. After title and abstract screening, 54 titles were pre-selected, from which 23 were finally included, after full-text reading and eligibility criteria was applied.

Table 1 shows the study variables of the bibliographic review. The articles that met the inclusion requirements were published between 2000 and 2017. In regard to the countries of origin, six articles came from Germany, three from the USA, two from Switzerland, United Kingdom and Poland, while Belgium, Canada, Italy, Australia, Serbia, Czech Republic and South Africa contributed one article each (column 1 of Table 1). Column 2 shows that the goal of most of the studies was to identify doping substances (substances/metabolites/markers) in dietary supplements. Six studies determined whether the intake of contaminated dietary supplements could result in a positive test in anti-doping controls. The characteristics of the sample of supplements or study subjects are shown in column 3. Column 4 identifies the tested substances/metabolites/markers that give positive results in anti-doping controls. Column 5 refers to the main results, and column 6 to the conclusions of the studies included in the review.

Regarding the number of samples selected, more than 100 supplements were analyzed, when considering all the articles incorporated in this review. In five of the studies included, it was the subjects who had taken the substances of interest that were analyzed. In 13 of the 23 articles, more than two contaminating substances were studied, while in three articles, two contaminants were studied and in seven articles there was only a single substance under study. The contamination rate found in studies where more than two ergonutritional supplements were analyzed, ranged from 12% to 58%. While nine of the 10 studies that analyzed one or two supplements had rates of contamination of 100%, in the study by Goel et al. [27], where a single supplement was analyzed, the results obtained showed no contamination. In one of the 23 studies, the metabolic effects, produced two hours after the ingestion of an ergonomic supplement, contaminated by 19-nor-4-androstenedione and 4-androsten-3,17-dione, were identified after the collection of urine samples, for a total of five individuals. In five of the 23 studies, banned substances were sought in three specific supplements, by analyzing urine samples.

The most commonly used methodology for the detection of any unidentified substance or one prohibited in ergonomic supplementation by any of the official bodies was gas chromatography coupled to mass spectrometry (GC-MS) (*n* = 10), followed by liquid chromatography coupled to mass spectrometry (LC-MS) (*n* = 3), combined GC-MS + LC-MS (*n* = 2), nuclear magnetic resonance (NMR) (*n* = 2), HPLC-DAD (*n* = 1), UHPL-MS/MS (*n* = 1) and the modified Geyer method (*n* = 1).

## 4. Discussion

Among the main findings of the present review is the presence of doping substances in studied dietary supplements or aids, which are not substances identified in the nutritional composition declared on the labeling, or whose amounts stated therein differ from their actual content. Among the substances found, but not listed, on the label are prohormones, anabolic steroids, mental enhancers, and 1,3-dimethylamylamine. All of these are substances that are prohibited by the WADA, which would give a positive doping test result for the athletes who have consumed these supplements or ergonutritional aids. Some of the studies analyzed the presence of contaminants in human subjects after the consumption of contaminated supplements or ergonutritional aids; in other studies, the products themselves were analyzed.

### 4.1. Consumption and Contamination of Ergonutritional Supplements

The consumption of ergonutritional supplements is one of the most common practices in the sports world; advertisements for such products claim that their use will prevent injuries or enhance performance [27]. They can be used by as many as 90% of participants, depending on the sport [28]. Linked to these high frequencies of consumption, we have found that one of the most serious and increasingly frequent problems regarding the intake of dietary supplements is unintentional doping. The consumption of these supplements forms part of the daily routine of most athletes, who must be completely sure of the efficacy and safety of any type of dietary supplement before its consumption, as well as of its detailed composition. The data reported by some studies are noteworthy; for instance, the rate of contamination in ergonutritional supplements varied from 12% to 58% in samples analyzed between 2002 and 2005, and in 216 cases, hormones were found in dietary supplements that should not have contained them [17,29,30]. To avoid this, the controls and legislative strategies related to these supplements need to be improved, to guarantee the safety of products that are freely available to the general population and athletes.

Specifically, in the present review, all the papers included showed the presence of substances that are prohibited by the WADA in some of the dietary supplements analyzed. The most frequently encountered components in these products were anabolic steroids (banned by the IOC since 1974 after the positives detected at the Commonwealth Games held in New Zealand), although other prohibited substances were also present—such as certain stimulants (ephedrine, nor-pseudoephedrine, sibutramine) [11,31,32]. In addition to the serious effects that the consumption of these contaminated substances can have on health—such as hepatotoxicity, cardiac and hormonal problems, carcinogenesis, and even death in some cases [4,31]—the following can be added: social damage, related to moral damage, loss of sponsors, and penalties (among others), deriving from possible detection in doping tests.

The presence of substances not listed on labeling and banned by the WADA is not the only problem derived from the consumption of supplements. The lack of precision in the labeling of these products, in terms of quantity, is another of the problems associated with the consumption of such substances, according to various studies [17,29,33,34,35].

This review of the literature indicated that the consumption of supplements occurs in a high percentage of athletes, mainly driven by coaches, relatives, and other athletes, with the aim of achieving better results. One of the most important studies regarding the consumption of supplements is the one made by Tscholl et al. in 2010 [9], in which the data of 3887 questionnaires were collected during the world championship of the International Association of Athletics Federations. This study showed the consumption of 6523 supplements (1.7 per athlete); the consumption was greatest in adults and in participants in outdoor competitions. A study of 567 Canadian athletes between the ages of 11 and 25 found daily intake of supplements by 28% of them, with the main goal being to improve the consumption of vitamins and minerals and to improve performance [36]. Another study, involving 292 Portuguese athletes, from 13 different federations, showed a consumption rate of 66%, with an average of four supplements per athlete, with acceleration of recovery (63%) and improvement of performance (62%) being the main reasons for consumption [45]. 

It was from the year 2000 when the problems caused by unintentional doping began to take on importance, and the first studies on the quality of nutritional supplements were carried out [17,46]. The contamination rate due to errors in labeling, either by omission of substances present in the product, or by errors in the quantification of the concentrations, is relatively high, according to the various studies carried out [4,10,12,46,47,48]. One of the most relevant studies, due to the number of supplements analyzed, which laid the groundwork for the determination of the contamination of nutritional supplements, is that performed by Geyer et al., in 2001, in Germany, where 634 non-hormonal supplements were analyzed in the search for testosterone and its prohormones, nandrolone and its prohormones, and boldenone [12]. The results showed that 15% of the samples contained hormones or prohormones that were not identified in the labeling. A similar study was conducted by Kamber et al., in 2001, in which the objective was the detection of anabolic steroids or stimulants, not indicated, or poorly described, on the label [17]. The study analyzed 75 products, of which 17 were prohormonal supplements, and all contained substances not described in the labeling. In 2004, a study was published, in which 103 supplements, purchased online, and divided into four categories (creatine, prohormones, mental enhancers, and branched-chain amino acids), were analyzed. In this case, the most common contaminant was testosterone and the products with the highest contamination rate were prohormones. The labeling error rate was 18%, whereas 20% of the products contained metabolites of different hormones not allowed by the WADA [49].

Many of the studies involving contamination in supplements are aimed at validating a precise method of analysis for the detection of compounds banned by entities, such as the World Anti-Doping Association. An example of this is the study by Martello et al., in which gas chromatography coupled to tandem mass spectrometry (GC–MS/MS) was used as a screening system to detect certain androgenic steroids and ephedrine in dietary supplements. Thus, 64 nutritional supplements, obtained from stores and by judicial procedures (and classified as four vitamins/mineral supplements, seven glutamine/creatine, nine amino acids, 12 protein, eight prohibited substances, 12 herbal extracts and four others) were analyzed. Through this method, anabolic steroids and ephedrine were detected in 12.5% of the analyzed samples [34]. 

Finally, the online expansion of the advertising and marketing of ergonutritional supplements for sportsmen and women on the Internet has begun to constitute a public health problem. This is due to the free sale of these products without the health authorities carrying out the necessary inspections of the distribution and marketing. A study published by Van Poucke in 2006 analyzed 19 dietary supplements obtained via the internet. Fifteen of these claimed, on the labeling, the presence of between one and five prohormones, but 11 supplements were suspected of containing at least one anabolic steroid. Liquid chromatography showed that all the suspect substances contained at least one anabolic steroid, with testosterone and b-boldenone being the banned substances with the highest rates of use [31]. 

As for the factors causing this contamination, there are two main causes: (1) cross-contamination and (2) intentional contamination. Cross-contamination occurs unintentionally, as described by Hon and Coumans, because the prohormone concentration is low, which would not produce a potentiating effect of the supplement [16]. This occurs mainly because the manufacturers of prohormones (sold legally as supplements in the United States until 2004) also make other nutritional supplements. Cross-contamination could be due to the lack of cleaning of the vitamin containers, since the same production line is used without sufficient cleaning of the machinery [4,10]. The consumption of supplements affected by cross-contamination, despite the low concentration of contaminants, can lead to cases of unintentional doping [17]. Intentional contamination occurs when high concentrations of prohormones are added to the supplement by the manufacturer, with the aim of enhancing its effects [46]. 

Geyer et al. analyzed the number of nutritional supplements subject to cross-contamination with prohormones in different countries, between 2001 and 2002. The United States and Germany were the countries with the highest production of supplements, although The Netherlands and Austria had the highest contamination rates in their products [48].

### 4.2. Anti-Doping Organizations

Because of this, mechanisms of action have been put in place to combat contamination in supplements. The purpose is to produce a reliable source of information in which the athlete can check the safety of the supplement to be consumed [50]. The WADA, one of the main bodies that deals with the detection and prevention of doping in athletes, has established a strict liability policy, which states that unintentional doping is the responsibility of the athlete. Therefore, even if the athlete had no intention of improving his/her performance through the use of prohibited substances, if a doping control proves positive due to the use of contaminated ergonutritional supplements, it is the athlete, not the manufacturer or the seller, on whom the established sanction would fall. To avoid this type of situation, the WADA publishes—via the internet—the novelties and adverse findings for the supplements analyzed by its accredited laboratories. Other entities, such as the Court of Arbitration for Sport (TAS) [51], make athletes aware of registered doping cases and provide information regarding the possible source of the prohibited substance. Contributions are also made by National Anti-Doping Organizations (NADOs), such as that of Australia (ASADA) [52]—which offers an online search tool (Global DRO) to athletes and support personnel, to find out whether the most commonly prescribed and over-the-counter medicines in Australia are permitted or prohibited in their sport. Two other organizations pursuing similar strategies are the UK Anti-Doping Authority (UKAD) [53] and the US Anti-Doping Agency (USADA) [54]. In addition, there are other ways to check the safety of ergonutritional supplements, unofficially and without being endorsed by the WADA or the respective NADO—such as the Anti-Doping Authority the Netherlands (NZVT) project in Holland [55], the Cologne List in Germany [56], Informed Sports in the UK [57], the NSF Certified for Sports program of the Canadian Center for Sports Ethics [58], the Drug-free Sport NZ application of the New Zealand Anti-Doping Agency [59], the Supplement 411 program of USADA [60], or the “Alerts” section of the website of the Spanish Agency for the Protection of Health in Sport (AEPSAD) [61].

### 4.3. Limitations

Some limitations of this review, inherent in the use of electronic searches and retrieval of documents, should be pointed out. One of the most important limitations is that not all papers included analyzed the same prohibited compounds neither the same kind of samples, so several prohibited substances not analyzed could be also present in those products.

## 5. Conclusions

The safety issue regarding dietary supplements is real and therefore an improvement of the current legislation regulating the market for dietary supplements is needed to ensure the safety, efficacy, potency, and legality of the available ergonutritional supplements. Hence, the awareness of both athletes and coaches of the possible consequences of the use of ergonutritional supplements is especially important, as are discussions of the advantages and disadvantages and the provision of information related to the safety, provenance, and effectiveness of any type of supplement, before its consumption. The use of supplements without a specific need, illness, or deficiency—in addition to not being recommended—is unnecessary, when the athlete is following a balanced and adapted diet. Despite the strategies implemented by different governmental agencies to avoid doping in athletes, some positive doping results might be non-intentional and caused by the consumption of dietary supplements contaminated with doping substances.

Likewise, the fact that, in these products, information is often omitted from the labeling is a reason for sanctioning the companies that manufacture these food substances—since they are providing inaccurate or incomplete data—in accordance with Spanish Law 28/2015, for the defense of food quality [62]. This shows non-compliance with food labeling legislation, intended to protect the quality, the regulator of which is the government.

Although our work shows the existence of several dietary supplements on sale containing prohibited substances, more comprehensive studies are needed to know the extent and the prevalence of this problem.

Therefore, the previously described factors that affect food quality could be considered as an avoidable public health problem that indicates the need for governments to establish control strategies for procedures throughout the food chain, to generate a high level of confidence in dietary supplements which are habitually consumed by athletes. Likewise, compliance with the general principle of veracity and demonstration of the information contained in the labeling of ergonutritional products, must be guaranteed.

## Figures and Tables

**Table 1:** Information on studies analyzing contamination with substances/metabolites/markers prohibited by the World Anti-Doping Agency (WADA) in ergonutritional supplements.

Author/Year/Country		Aim	Sample	Selected Markers	Main Results	Conclusions
Van Thuyne., et al., 2006 [5]	South Africa	Determine whether the intake of contaminated dietary supplements can make anathlete positive in an anti-doping test.	5 male volunteers (24–55 years old) (11–99.4 kg)	19-nor-4-androstenediona y 4-androsten-3,17-diona	All exceeded the minimum amount established by WADA 2 h post-intake. Two exceeded the minimum amount 36 h post-intake. The maximum value was 54.6 ng/mL (8 h post)	The intake of only micrograms of contaminated substance can provoke a positive in an antidoping test
Geyer, H., et al., 2004 [12]	Germany	Analysis of 634 non-hormonal supplements to identify possible contamination of undeclared prohormones.	634 supplements	Testosterone and its prohormones, nandrolone and its prohormones and baldonone	Of the 634 supplements analyzed, 94 presented unidentified contaminants on their labeling	Despite offering guarantees in terms of pollutants, the population must be cautious about using ergonutricional substances, since not all these products are free of doping substances.
Green, H., et al., 2001 [16]	EEUU	Determine if steroids in supplements meet the Dietary Supplement Health and Education Act (DSHEA) labeling laws.	12 prohormones from 12 different brands, purchased from local stores.	5-androstenediol, 5-androstene-3,17-dione, 5-androstene-3b, 17b-diol, 4-androstene-3,17-dione, 5-androstene-3,17-diol, 19- Androstene-3b, 17b-diol, 4-androstene-3,17-diol, 19-nor-4-androstene-3,17-dione, 19-norandrostenedione, androstenedione, 19-nor-5-androstene-3,17-diol, Tribulus terrestris	Authors found that 11 of 12 brands tested did not meet the labeling requirements set out in the 1994 Dietary Supplement Health and Education Act. One brand contained 10 mg of testosterone, a controlled steroid, another contained 77% more than the label stated, and 11 of 12 contained less than the amount stated on the label.	The current study validates the concerns of physicians and sporting organizations that the labeling of some sports nutritional supplements does not accurately reflect what is contained in the product. This information may be helpful in deterring athletes from using substances that have unsubstantiated efficacy and unknown adverse effects.
Kamber, M., et al., 2001 [17]	Switzerland	Determine whether the products tested contain anabolic steroids or stimulants not indicated or poorly described on the label.	75 products	Anabolic steroids or stimulants not listed or poorly described on labeling	In 7 out of 17 prohormones, different substances than indicated on the labels were found. This corresponds to 41% of the products in this class of supplements and 9% of all analyzed supplements. In two other products (“mental enhancers”), caffeine and ephedrine were found. Both compounds were either not, or not clearly declared, on the labels (e.g., declaration of the plant Ma Huang that contains ephedrine). The concentration of ephedrine in product 56 was so high that an athlete would test positive for doping if only one capsule was consumed just before competition.	It is recommended that athletes use only supplements that are registered in Switzerland (and even these supplements may not be entirely free of contaminates). In light of the easy availability of medicines and nutritional supplements through the Internet, we should strive to inform and educate users (especially adolescents) about nutritional supplements, and support international standards for accurate product labeling.
Goel, D.P., et al., 2004 [27]	Canada	Address and determine the feasibility of conducting clinical tests on a dietary supplement (Cold-FX^®^) under strict International Olympics Committee (IOC) doping-control procedures and to determine whether ingesting this ginseng extract would result in any doping-control infractions among athletes.	20 men and 20 women	Ginseng Extract (Cold-FX)	No positives were found for prohibited substances in any of the subjects′ urine samples.	Cold-FX^®^ substance is safe. This work could encourage companies to test dietary supplements so that athletes and athletic regulatory bodies have access to competent, comprehensive, credible, and unbiased information on the capacity for nutraceuticals and dietary supplements to induce positive urinalysis tests.
Baume, N., et al., 2006 [28]	Switzerland	To screen the supplements for con-taminations with major anabolic steroid parent compounds, stimulants and traces of testosterone, nandrolone and their precursors	103 supplements, divided into creatine, prohormones, mental enhancers and branched amino acids, all purchased online.	4-Androstenediol, 4-Norandrostenediol, 5-Androstenedione, 5-Norandrostenediol, 19-Noranrostenedione, Androstenediol, Androstenedione, Bolasterone, Boldenone, Clostebol, Dehydroepiandrosterone (DHEA), Dihydrotestosterone (DHT), Drostanolone, Fluoxymesterone, Mesterolone, Metandienone, Metenolone, Methyltestosterone, Norethandrolone, Oxandrolone, Oxymesterone, Stanozol, Oxymetholone, Nandrolone, Testosterone, Testosterone Propionate, Turinabol, 5-Norandrostenedione.	Methandienone was found in 3 of the 103 products. 18% of the products had errors in the labeling, while 18 products were found to contain metabolites of testosterone or nandrolone. The most commonly used contaminant was testosterone and the most contaminated product was prohormonesl.	More studies are needed to analyze contamination in products or poorly labeling, in order to prevent and improve the quality of dietary supplements available in the market.
Martello, S., et al., 2007 [29]	Italy	Validation of a qualitative LC-MS/MS method for the determination of eight doping substances.	64 supplements obtained from stores and court proceedings.	4-androsten-3,17-dione, 4-oestrene-3,17-dione, 5α-androsten-17β-ol-3-one, Boldenone, Nandrolone, Nandrolone Decanolate, Testosterone, Testosterone Decanolate, Ephedrine	This LCMS/MS method was applied to 64 nutritional supplements and 12.5% of tested substances contained prohibited substances (anabolic steroids and ephedrine) not stated on the labeling.	The method reported is sufficiently sensitive, specific and selective for the detection and confirmation of prohibited substances in nutritional supplements. The low levels of the compounds found in the samples may indicate accidental contamination and not intentional admixture. However, athletes should consider only purchasing from companies that perform quality tests on prohormones, and which test for possible contamination during production.
Parr, M. K., et al., 2008 [30]	Germany	Detection of clenbuterol in a sample of a fat burner	Sample of urine, 3 h post ingestion of a supplement tablet	Clenbuterol	After ingesting one tablet the participant reported tremor and delivered a urine sample. This urine was found to contain 2 ng/mL of clenbuterol utilizing LC-MS/MS analysis. Additionally the product itself was analyzed with gas chromatography coupled to mass spectrometry (GC-MS) for clenbuterol, yielding a content of about 30 µg per tablet.	The beta-2 agonist clenbuterol is only legally available on prescription and is classified as a prohibited doping substance in sports. The present case, for the first time, confirms the presence of clenbuterol in a dietary supplement. It again demonstrates the common problem with products on the supplement market, where non-licensed pharmaceuticals and doping substances are easily available. The ingestion of these products, containing additions of therapeutic drugs, can lead to side effects and/or interactions with conventional medicines.
Van Poucke, C., et al., 2007 [31]	Belgium	Determination of anabolic steroids in dietary supplements.	19 dietary supplements obtained via the internet from 12 different companies.	Α and β Boldenone, α and β Nortestosterone, 17α-Hydroxyprogesterone, Algeston acetophenide, Chloromadinon Acetate, Clostebol Acetate, Delmadinone Acetate, Fluoximesterone, Formeblone, Megestrol Acetate, Melengestrol Acetate, Methylboldenone, Methyltestosterone, Norethandrolone, Noretistestosterone, Norgestrel, Oxymetolone, Progesterone, Stanozol, Trenbolone, α and β Zeranol, D-equilenin, Dienestrol, Diethylstolbestrol, Ethinyl estradiol, Estradiol, Hexestrol, Testosterone, 16-dehydroprogesterone, 17α-acetoxyprogesterone, tenbolon 17β-acetate, 20β-hydroxyprogesterone, 3α and Β-hydroxy-5β-estrane-17-one, α-testosterone, ethylstiltranediol, Flugestonacetate, Medroxyprogesterone acetate, Mestranol, Metandriol, Metenolone, Methenolone acetate, Methylandrantranediol, Norethystostosterone acetate, Noretilnodrel, Vinylstestone.	According to the labeling, 15 of the 19 products contained 1–5 prohormones. Eleven products contained at least one anabolic component, all of these products claimed to contain prohormones.	The analysis of the 19 dietary supplements, indicates that the supplements named are not suitable for athletes. In addition to having prohormones that can be activated in the body, anabolic steroids were found in their active form.
Parr, M.K., et al., 2011 [32]	Germany	Identification of Δ6-methyltestosterone in a product named “Jungle Warfare“, which was obtained from a web-based supplement store	1 subject (52 years, 77 kg, 1.70 m)	(Epi-) methyltestosterone	The presence of the study metabolite was confirmed both in the analysis of the supplement and in the urine sample of the study subject.	The Jungle Warfare supplement represents another product labeled as a dietary supplement that contains steroids not approved for medical use.
Watson, P., et al., 2009 [33]	UK	To detect urinary excretion of nandrolone metabolites after ingestion of a precursor of nandrolone.	20 subjects (11 men and 9 women) recreational athletes	19-norandrostenedione (nandrolone)	With the intake of a supplement contaminated with 1 μg, no athlete would give a positive result in a doping control. In the case of 2.5 μg, 5 subjects would give a positive result and 15 subjects, would pass the minimum level (2 ng/mL) allowed, giving a positive result in the test of 5 μg of nandrolone.	Ingestion of trace amounts of 19-norandrostenedione can result in transient elevations of urinary 19-NA and 19-NE concentrations. The addition of as little as 2.5 kg of 19-norandrostenedione to a supplement (0.00005% contamination) appears sufficient to result in a doping violation in some individuals.
Parr, M.K., et al., 2007 [34]	Germany	Check the lack of safety in the production of supplements and obtaining supplements.	2 dietary supplements were analyzed (Stanozol-S and Parabolon-S) obtained by telephone.	Methandienone, norandrostenedione, stanozolol, testosterone, 5α-dihydrotestosterone, boldenone and estrone.	In Parabolon-S, metandienone was found. In addition, Stanozolol-S, stanozol, testosterone, 5α-dihydrotestosterone and boldenone were found.	There is insufficient surveillance of the production and trade of dietary supplements. Consumers should be aware of the enormous health and doping risks connected with the use of such products. New regulations for trade, production and labeling should be adopted. The first step should be a public warning to consumers and the withdrawal of dietary supplements containing prescription drugs.
Thevis, M., et al., 2013 [35]	Germany, EEUU and Switzerland	Study the ability to detect the origin of clenbuterol (therapeutic use or food intake) depending on the presence of racemic mixtures (enantiomers).	6 urine samples, collected from 2 male subjects	Stereoisomers + and – of Clenbuterol		The determination of relative abundances of clenbuterol enantiomers can indicate the ingestion of clenbuterol via contaminated food; however, depletion of (-)-clenbuterol in edible animal tissue is time-dependent and thus results can still be inconclusive as to the inadvertent ingestion of clenbutero, l when clenbuterol administration to animals was conducted until slaughter.
Monakhova, Y.B., et al., 2014 [36]	Germany	Test an magnetic resonance (NMR)-based method with minimal sample preparation for determination of 1,3-dimethylamylamine (DMAA) in sports nutrition and dietary supplements.	16 sports nutrition products and dietary supplements	1,3-dimethylamylamine	9 of the 16 substances were contaminated with DMAA.	Routine application of NMR is an alternative to time-consuming chromatographic methods for DMAA quantification in various kinds of products. 1H NMR spectroscopy has proven to be a robust analytical tool, yielding highly reliable quantitative results regarding DMAA, in a very short time. The approach is advantageous as it minimizes sample preparation and allows for the analysis of a large number of samples without human intervention (120 samples in a batch). The developed NMR method is recommended for use in food testing, customs and doping control laboratories, for the routine control of DMAA.
Abbate V., et al., 2014 [37]	United Kingdom	Determine any steroid present in the supplements, using full scan gas chromatography-mass spectrometry (GC-MS), accurate mass liquid chromatography-mass spectrometry (LC-MS), high pressure liquid chromatography with diode array detection (HPLC-DAD), UV-Vis, and nuclear magnetic resonance (NMR)	A total of 24 products were purchased from two fitness equipment shops—one in Merseyside and one in Cheshire—and three online shops.	Anabolic steroids	Of the 24 products tested, 23 contained steroids, including known anabolic agents; sixteen of these contained steroids that were different to those indicated on the packaging and one product contained no steroids at all. Overall, 13 different steroids were identified; 12 of these are controlled in the UK under the Misuse of Drugs Act 1971. Several of the products contained steroids that may be considered to have considerable pharmacological activity, based on their chemical structures and the amounts present. This could unwittingly expose users to a significant risk to their health, which is of particular concern for naïve users.	The analytical methods used can play an essential role in the public health response to these drugs by providing methodologies to identify and quantify the active substance(s) present. This helps develop our understanding of this market, as well as allowing us to monitor the composition of supplements sold and the hazards that they may pose. When considered with other data, such as prevalence of use, these types of study play a central role n assessing and quantifying the risks to individual and public health.
Cooper E.R., et al. 2017 [38]	Australia	Characterize the androgenic bioactivity of sports supplements available from the Australian market, using yeast and mammalian cell androgen bioassays.	112 sports supplements available from the Australian market, either over the counter or via the Internet.	Androgens (Androgen bioactivity)	All 112 products did not declare an androgen on the label as an included ingredient. Our findings show that 6/112 supplements had strong androgenic bioactivity in the yeast cell bioassay, indicating products spiked or contaminated with androgens. The mammalian cell bioassay confirmed the strong androgenic bioactivity of 5/6 positive supplements. Supplement 6 was metabolized to weaker androgenic bioactivity in the mammalian cells. Further to this, supplement 6 showed a positive result in a yeast cell progestin bioassay.	These findings highlight that nutritional supplements, taken without medical supervision, could expose or predispose users to the adverse consequences of androgen abuse. The findings reinforce the need to increase the awareness of the dangers of nutritional supplements and highlight the challenges that clinicians face in the fast-growing market of nutritional supplements.
Cohen E.A., et al., 2013 [39]	USA	Detect the presence and concentration of N,alpha-Diethylphenylethylamine (N,α-DEPEA) in supplement Craze (Driven Sports, Inc.)	Three samples from different lot numbers of Craze	N,α-DEPEA	The identity of N,α-DEPEA was confirmed using nuclear magnetic resonance and reference standards. Manufacturer recommended servings were estimated to provide 21 to 35 mg of N,α-DEPEA. N,α-DEPEA has never been studied in humans. N,α-DEPEA is a methamphetamine analog; however, its stimulant, addictive and other adverse effects in humans are entirely unknown.	If the findings are confirmed by regulatory authorities, the Food and Drug Administration (FDA) should take immediate action to warn consumers and remove all N,α-DEPEA-containing supplements from the marketplace.
Stepan R., et al., 2008 [40]	Czech Republic	Analytical approach employing ethyl acetate extraction, dispersive solid-phase extraction (SPE) clean-up using PSA followed by an analysis of underivatized compounds, using comprehensive two-dimensional gas chromatography with time-of-flight mass spectrometric detection (GCxGC-TOF MS) is presented.	Two types of commercially available solid nutritional supplements: protein concentrate and creatine monohydrate	Anabolic steroids	Results from this monitoring programme showed a total 6.3% (i.e., three) positive samples. Nandrolone (0.022 mg kg^–1^), testosterone (0.070 mg kg^–1^) and DHEA (0.063 mg kg^–1^) were found in a whey protein gainer, 5-androstan-3,17-dione (0.398 mg kg^–1^) and 19-norandrostendione (0.304 mg kg^–1^) in creatine pyruvate, and one sample of synephrine-based ‘fat burner’ contained progesterone (0.102 mg kg^–1^).	This analytical method, based on the comprehensive two-dimensional gas chromatography with timeof-flight mass spectrometric detection, provides an advantageous strategy for the determination of anabolic androgenic steroids and related compounds in nutritional supplements. The use of dispersive solid-phase extraction (SPE) with primary secondary amine (PSA) for the clean-up of crude extracts prepared from other matrices could likely be an efficient process for removing interferences and should be considered individually, according to the type of co-extracted matrix components.
Parr M.K., et al., 2011 [41]	Germany and Poland	Warn about the presence of designer steroids in some dietary or nutritional supplements.	Reports a few doping cases caused by the use of supplements with doping substances by athletes, noted by two World Anti-Doping Agency (WADA) accredited laboratories (Cologne and Warsaw)	Steroids	Steroids that may be interpreted as metabolites of Δ6-methyltestosterone, were detected. The availability of the athletes‘ supplements allowed for confirmation of this interpretation, as one of the products indeed contained Δ6-methyltestosterone. These findings confirmed the presumption that such products are used by athletes and that their consumption may lead to positive results in doping controls.	Top level athletes use “dietary supplements” that contain so-called designer steroids. The statistics of the World Anti-Doping Agency in recent years has reported some more cases with steroids that are only available in dubious products and not as approved pharmaceuticals. However, people outside of elite sport were also found to have used such designer supplements. Still more education on the health and doping risks of dietary supplement products seems to be necessary for the protection of both athletes and the general public.
Stajic A., et al., 2017 [42]	Serbia	Develop and validate the sensitive and reliable ultra-high pressure liquid chromatography tandem mass spectrometry (UHPLC/MS/MS) method for determination of higenamine in different dietary supplements.	Different dietary supplements of various compositions and pharmaceutical forms were collected. Among all collected supplements, 19 were of interest for higenamine analyses. Dietary supplements were purchased in sport shops, via the Internet or from the local pharmacy. Samples were taken from the original packages, adequately labeled and stored.	Higenamine	According to the results, most of the investigated supplements were free of higenamine, but on the other hand, the presence of higenamine was confirmed in some samples, while it was not declared on the label. Presence of higenamine, a banned substance, was confirmed in two investigated samples.	A sensitive and reliable UHPLC/MS/MS method for higenamine determination in various dietary supplements was developed and validated. This method was successfully applied for the analysis of 19 dietary supplements and, in this way, applicability of the method was confirmed.
Kohler M., et al., 2010 [21]	Germany	Provides an overview the products that were analyzed in the Cologne Doping Control Laboratory in 2009 and gives an overview on the classes of substances and the astonishingly small number of products that contain exactly the labelled substance.	A number of different products were analyzed from various sources, such as customs, police, and national anti-doping authorities, or were bought over-the-counter as nutritional supplements. Most of the commodities contained protein- or peptide-based substances, many of which were not in agreement with their respective labels or contained poorly purified analogues or artefacts.	Long-R3-IGF-I, GHRP-2, Andarine (S-4)	The products analyzed during 2009 showed that black market products nowadays also include different peptide hormone-derived products rather than steroid hormone preparations only. From the confiscated products, only 4 out of 11 contained the substance and amount declared on their label, and long- growth factor 1 (R3-IGF-I) and human growth hormones were the proteins detected (or at least labelled) most frequently (three products each), which may indicate that they are also ordered and used very often. In contrast, the nutritional supplements containing Growth Hormone releasing Peptide-2 (GHRP-2) as well as the glass bottle with Andarine (S-4) were labelled with xenobiotic ingredients, although none of them are approved as regular therapeutic agents yet.	The awareness of new products on the black market and in nutritional supplements is of utmost importance for laboratories to develop detection methods accordingly and screen for new substances as early as possible.
Thomas A., et al., 2010 [43]	Germany	The qualitative identification and quantification of the approximate content of GHRP-2 in tablets offered as nutritional supplements by means of liquid chromatography coupled to high resolution/high accuracy mass spectrometry, is described.	Nutritional supplements in tablets	GHRP-2 and Hexarelin		The presented case report demonstrates the urgency of flexible analytics in doping controls. Although, to date no positive doping cases with GHRP-2 were reported, the fact that the bioactive compound is available as a nutritional supplement, indicates that analytical findings in routinely analyzed plasma or urine samples from elite sportsmen are possible.
Kwiatkowska D., et al., 2015 [44]	Poland and Australia	Analyze urine samples to detecte stimulants and narcotics in anti-doping controls	The urine samples were taken from four athletes during in competition anti-doping control, and nutritional supplement NOXPUMP Pre-Training Formula (Fruit Punch)	Stimulants and narcotics	N,N-dimethyl-2-phenylpropan-1-amine (NN-DMPPA) is a possible new doping agent, detected and identified by the WADA-accredited laboratory in Warsaw (Poland) during routine anti-doping control. The presence of NN-DMPPA in several urine samples and in the supplement, NOXPUMP, was confirmed by GC-MS. In most of the athletes who failed urine drug tests because of the presence of NN-DMPPA, some other banned stimulants were also detected. NN-DMPPA was detected in the supplement NOXPUMP but we cannot exclude its present in other supplements from the black market.	This suggests the use of supplements which are often mixtures of prohibited drugs which may not be listed on the label or even the use of several supplements containing various banned substances. The range of supplements available in stores is constantly growing and while many supplements contain materials (e.g., vitamins, proteins, minerals) with possible useful properties, many pose the risk of unintentional doping with designer agents. This is often caused by the lack of labelling of all contents and/or unusual naming of components on the supplement label. Improved legistation, dealing with the commercialization of the drugs banned for sport, should be enacted.

## Abbreviations

NSAIDs	Nonsteroidal anti-inflammatory drugs
WADA	World Anti-Doping Agency
IOC	International Olympic Committee
TAS	Court of Arbitration for Sport
NADO	National Anti-Doping Organization
ASADA	Australian Sports Anti-Doping Authority
UKAD	UK Anti-Doping Authority
USADA	United States Anti-Doping Agency
NZVT	Anti-Doping Authority the Netherlands
AEPSAD	Spanish Agency for Health Protection in Sport
DHEA	Dehydroepiandrosterone
DHT	Dihydrotestosterone
NMR	Magnetic resonance
N,α-DEPEA	N,alpha-Diethylphenylethylamine
SPE	Solid-phase extraction
PSA	Primary secondary amine
UHPLC /MS/MS	Ultra-high pressure liquid chromatography tandem mass spectrometry
R3-IGF I	Growth factor 1
GHRP-2	Growth hormone releasing peptide-2
S-4	Andarine
